# Magnitude and Factors Associated with HIV Viral Suppression Among Adult People Living with HIV-HBV Co-Infection in Northwest Ethiopia

**DOI:** 10.3390/tropicalmed11070175

**Published:** 2026-06-26

**Authors:** Mequanente Dagnaw, Destaw Fetene Teshome, Tilahun Bizuayehu Demass, Abebaw Gebyehu Worku

**Affiliations:** 1 Department of Epidemiology and Biostatistics, Institute of Public Health, University of Gondar, Gondar P.O. Box 196, Ethiopia; 2 Department of Medical Biotechnology, Institute of Biotechnology, University of Gondar, Gondar P.O. Box 196, Ethiopia; 3 Department of Internal Medicine, College of Medicine and Health Sciences, University of Gondar, Gondar P.O. Box 196, Ethiopia; 4 Department of Reproductive Health, Institute of Public Health, College of Medicine and Health Sciences, University of Gondar, Gondar P.O. Box 196, Ethiopia

**Keywords:** viral suppression, antiretroviral therapy, medication adherence, HIV-HBV, Ethiopia

## Abstract

**Background:** HIV-HBV co-infection remains a major public health challenge, particularly in sub-Saharan Africa. HBV co-infection worsens clinical outcomes among people living with HIV by accelerating liver disease and complicating treatment. Although antiretroviral therapy can effectively suppress both viruses, achieving optimal HIV viral suppression remains critical for reducing morbidity and transmission. While several factors influencing viral suppression among PLHIV are well documented, evidence on HIV viral suppression among HIV-HBV co-infected individuals is limited, especially in resource-limited settings like Ethiopia. Furthermore, data on the magnitude of viral suppression and its associated factors in this population are scarce. Therefore, this study aimed to assess the magnitude of HIV viral suppression and identify its associated factors among adult HIV-HBV co-infected patients in Northwest Ethiopia. **Objective:** This study aimed to assess the magnitude and factors associated with HIV viral suppression among adult people living with HIV-HBV Co-infection in Northwest Ethiopia. **Methods:** An institution-based cross-sectional study was conducted in Northwest Ethiopia among adults with HIV-HBV co-infection on antiretroviral therapy. A simple random sample of 402 participants was selected. Data were collected using a pretested structured interviewer-administered questionnaire and medical record review, covering sociodemographic, clinical, behavioral, treatment, follow-up, and adherence factors. **HIV** viral suppression was defined as a plasma viral load < 1000 copies/mL. Data were coded in EpiData 4.6 and analyzed using STATA 18. Descriptive statistics estimated suppression rates. Bivariable and multivariable logistic regression identified factors associated with suppression; variables with *p* < 0.25 in bivariable analysis were included in the multivariable model. Statistical significance was set at *p* < 0.05 with adjusted odds ratios and 95% confidence intervals reported. Model fit was assessed using the Hosmer–Lemeshow test, and multicollinearity was checked using variance inflation factors. **Results:** There were 423 participants in all. Among the 423 HIV-HBV co-infected adults on antiretroviral therapy included in this study, 138 (34, CI, 30–39%) achieved HIV viral suppression, while 264 (66%) had an unsuppressed viral load at the time of assessment. Viral suppression was found to be independently correlated with the ART TDF-3TC-LPV/r regimen, first-line medication adherence, bedridden functional level, missed clinic appointments, and length of therapy. While TDF-3TC-LPV/r usage (AOR 2.34; 95% CI: 1.40–3.90) and longer treatment duration (AOR 2.09; 95% CI: 1.30–3.34) were advantageous, good adherence significantly improved the likelihood of suppression (AOR 5.54; 95% CI: 3.27–9.38). Missed appointments and a bedridden state decreased the likelihood of suppression. **Conclusions:** HIV viral suppression was achieved in only 34% of participants. Adherence, ART regimen, treatment duration, functional status, and retention in care were significant predictors. Strengthening adherence support, patient retention, optimized ART regimens, routine viral load monitoring, and targeted care for high-risk patients could improve treatment outcomes and help Ethiopia achieve UNAIDS viral suppression targets.

## 1. Background

Human immunodeficiency virus (HIV) remains a major global public health concern, with an estimated 39 million people living with HIV (PLHIV) worldwide in 2023 [1]. Despite remarkable progress in scaling up antiretroviral therapy (ART), co-infections continue to pose significant challenges to treatment outcomes. Among these, hepatitis B virus (HBV) co-infection is particularly important due to shared routes of transmission, including sexual contact, blood exposure, and vertical transmission. Globally, approximately 5–10% of PLHIV are co-infected with HBV, with a disproportionately higher burden in sub-Saharan Africa where HBV is endemic [2,3].

HIV-HBV co-infection is associated with more rapid progression of liver disease, increased risk of cirrhosis and hepatocellular carcinoma, and higher mortality compared to mono-infection [4,5]. The introduction of ART, particularly tenofovir (TDF)-based regimens, has significantly improved outcomes because of their dual activity against both HIV and HBV [2]. Achieving viral suppression is the cornerstone of HIV treatment, as it improves immune recovery, reduces morbidity and mortality, and prevents onward transmission. This is central to the global UNAIDS 95-95-95 targets, which aim for 95% of individuals on ART to achieve viral suppression [6].

In sub-Saharan Africa, which accounts for nearly two-thirds of the global HIV burden, the coexistence of HIV and HBV presents a substantial clinical and public health challenge [6]. Health systems in the region often face constraints such as limited diagnostic capacity, shortages of trained healthcare providers, and inconsistent access to viral load testing, all of which can hinder optimal patient management [7,8].

In Ethiopia, the government has made considerable progress in expanding ART coverage and improving HIV care through national programs aligned with global strategies. According to national reports, Ethiopia has achieved significant gains in ART uptake and viral load monitoring; however, viral suppression remains suboptimal in certain populations, particularly among patients with co-infections and those facing adherence challenges [9]. The burden of HBV co-infection among PLHIV in Ethiopia further complicates treatment and necessitates integrated care approaches. HIV and hepatitis B virus (HBV) co-infection is a significant public health concern in Ethiopia, particularly in Northwest Ethiopia, where both infections remain endemic. Because HIV and HBV share similar routes of transmission, including sexual contact, exposure to contaminated blood, and vertical transmission, co-infection is common among people living with HIV (PLHIV). Evidence suggests that HBV co-infection accelerates liver disease progression, increases the risk of cirrhosis and hepatocellular carcinoma, and may negatively affect HIV treatment outcomes, including viral suppression and immune recovery [10]. A systematic review and meta-analysis in Ethiopia reported that the pooled prevalence of HIV/HBV co-infection among PLHIV was approximately 5.5%, indicating a substantial burden of disease and the need for integrated management strategies [11]. In Northwest Ethiopia, studies conducted at the University of Gondar and other health facilities have documented a considerable prevalence of HBV infection among HIV-infected adults, highlighting the importance of monitoring treatment outcomes in this population [10]. Despite the scale-up of antiretroviral therapy (ART) and routine viral load monitoring in Ethiopia, evidence regarding the magnitude of HIV viral suppression and factors associated with successful suppression among HIV/HBV co-infected adults remains limited. Therefore, assessing HIV viral suppression and its determinants among adults living with HIV/HBV co-infection in Northwest Ethiopia is essential to improve patient management and support the achievement of national and global HIV treatment targets.

Despite advancements in ART availability and effectiveness, achieving and sustaining viral suppression among HIV-HBV co-infected individuals remain challenging. Co-infection introduces clinical complexity, as treatment must effectively suppress both viruses while minimizing toxicity and resistance. Although TDF-based regimens are recommended, not all patients receive optimal therapy due to delayed diagnosis, regimen limitations, or programmatic constraints [2].

Globally, approximately 76% of individuals receiving ART achieve viral suppression, indicating that a substantial proportion still experience virological failure [6]. Factors contributing to this gap include poor adherence, treatment interruptions, drug resistance, and inadequate follow-up [12,13]. In co-infected individuals, these challenges are amplified due to the need for strict adherence to maintain suppression of both HIV and HBV.

In sub-Saharan Africa, barriers such as poverty, stigma, transportation difficulties, and health system inefficiencies significantly affect retention in care and adherence, leading to suboptimal viral suppression [7]. Missed clinic appointments, delayed switching of failing regimens, and limited access to routine viral load monitoring further contribute to poor outcomes.

In Ethiopia, although ART services have expanded widely, several studies report that not all patients achieve viral suppression, particularly those with poor adherence, advanced disease stage, or inconsistent follow-up [9,14]. Additionally, the management of HIV-HBV co-infection is challenged by limited routine screening for HBV, inadequate monitoring of liver function, and gaps in integrated care services. These issues highlight the need for more focused research on co-infected populations.

A substantial body of evidence has identified multiple factors influencing viral suppression. At the global level, adherence to ART is consistently the strongest predictor of virological success, with optimal adherence (≥95%) significantly increasing the likelihood of viral suppression [12,13]. Other important determinants include type of ART regimen, duration of treatment, baseline clinical status, and retention in care [6,15]. For HIV-HBV co-infected patients, TDF-based regimens combined with lamivudine (3TC) or emtricitabine (FTC) are recommended due to their dual antiviral activity [15].

In sub-Saharan Africa, studies emphasize the role of health system factors, including access to care, quality of services, and availability of viral load testing, in shaping treatment outcomes [7]. Socioeconomic factors, stigma, and patient mobility also indirectly affect viral suppression through their impact on adherence and retention.

In Ethiopia, several studies have identified key predictors of viral suppression, including good adherence, longer duration on ART, use of effective regimens, and consistent clinic attendance [9,14,16].

Furthermore, while some behavioral and social factors (e.g., stigma, disclosure, and social support) are known to influence adherence, their independent effect on viral suppression after adjustment remains inconsistent across studies. The interaction between HBV co-infection and HIV treatment outcomes, including its effect on viral suppression, is also not well understood in resource-limited settings. However, important gaps remain. There is limited evidence focusing specifically on HIV-HBV co-infected populations, particularly at regional levels such as Northwest Ethiopia. The magnitude of viral suppression and the relative contribution of clinical, behavioral, and health system factors in this group are not well characterized. Additionally, the interaction between HBV co-infection and HIV treatment outcomes, including its impact on viral suppression, is still underexplored in low-resource settings. Addressing these gaps is essential for designing targeted interventions and improving patient outcomes. Therefore, there is a critical need for context-specific evidence on the magnitude of viral suppression and its associated factors among HIV-HBV co-infected adults. Generating such evidence in Northwest Ethiopia will help inform targeted interventions, improve integrated care strategies, and contribute to achieving national and global HIV treatment goals. This study aims to assess the magnitude of viral suppression and associated factors among adult HIV-HBV co-infected patients in Northwest Ethiopia, providing evidence to inform clinical practice and public health strategies.

## 2. Methods

### 2.1. Study Design and Period

An institution-based cross-sectional study design was employed to assess the magnitude and associated factors of HIV viral suppression among adults living with HIV-HBV co-infection in North-West Ethiopia referral hospitals from 13 November 2025 to 12 January 2026.

### 2.2. Study Setting

This study was conducted in four selected referral hospitals of Northwest Ethiopia, located in the Amhara Regional State. These, Tibebe Gion Referral Hospital, Felege Hiwot Referral Hospital, Debre Tabor Referral Hospital, and University of Gondar Comprehensive Specialized Referral Hospital serve as major tertiary healthcare centers for large catchment populations from urban and rural areas, including surrounding zones and districts. The selected referral hospitals provide comprehensive HIV care and treatment services, including antiretroviral therapy (ART) initiation and follow-up, HIV viral load testing, HBV screening (HBsAg), clinical management of co-infections, and routine monitoring of treatment outcomes. They also offer laboratory services such as CD4 testing, liver function tests (ALT/AST), and in some settings, HBV DNA testing.

These hospitals follow the national HIV treatment guidelines of Ethiopia and provide integrated HIV care alongside other specialized medical services. At the time of the study, the referral hospitals were managing a large number of adults living with HIV, including a significant magnitude with confirmed HIV-HBV co-infection. The study included all eligible HIV-HBV co-infected adult patients receiving ART follow-up services at the selected referral hospitals during the study period.

### 2.3. Source and Study Population

#### 2.3.1. Source Population

The source population for this study consisted of all adult HIV-HBV co-infected patients aged 18 years and above who were enrolled in antiretroviral therapy (ART) follow-up care in selected four referral hospitals of Northwest Ethiopia referral hospitals from 13 November 2025 to 12 January 2026.

These patients were receiving routine HIV care and treatment services, including clinical follow-up, laboratory monitoring, and antiretroviral therapy as part of the national ART program.

#### 2.3.2. Study Population

The study population included all eligible adult HIV-HBV co-infected patients who were selected from the source population and met the inclusion criteria. Specifically, the study population consisted of HIV-HBV co-infected adults receiving ART follow-up care at the selected referral hospitals in Northwest Ethiopia during the data collection period, who: were aged ≥18 years, had confirmed HBV co-infection (HBsAg positive), had been on ART for at least 6 months, and had complete and accessible clinical and laboratory records. A total of 402 participants met the eligibility criteria and were included in the study, whereas 21 participants were excluded because of non-response.

### 2.4. Sample Size and Sampling Procedures

#### 2.4.1. The Sample Size Determination

The sample size for this study was determined using the single proportion formula based on the estimated magnitude of viral suppression/treatment success among HIV-HBV co-infected adults:

The sample size was calculated utilizing the single proportion formula, based on the assumptions of a 95% confidence interval (*z*), a 5% margin of error (*d*), and an estimated magnitude of 50%, as established by the rule of thumb [17].

$$n=\frac{\left({Z_{a/2})}^{2}p(1-p\right)}{d2}$$

where

Z_a/2_ = 1.96 is the critical point for the standard formal table

a = level of significance

n = sample size

d = 5% of margin of error $n=\frac{3.8416\times 0.5(1-0.5)}{{\left(0.05\right)}^{2}}$ = 384

A 10% non-response rate was added to compensate for incomplete records or non-participation, giving a final sample size of 423 participants.

#### 2.4.2. Sampling Procedures

The study was conducted in selected referral hospitals providing ART services in Northwest Ethiopia. Referral hospitals were selected using a simple random sampling technique. Within each selected hospital, a systematic random sampling method was employed to select study participants. A sampling interval of 2 was used, whereby after randomly selecting the first eligible participant, every second patient was included until the required sample size was reached.

### 2.5. Eligibility Criteria

#### 2.5.1. Inclusion Criteria

The study included adults aged 18 years and above with confirmed HIV-HBV co-infection. Eligible participants were those who had been receiving antiretroviral therapy (ART) for at least 6 months prior to data collection, as this duration is generally considered sufficient for assessing initial treatment response, including virological suppression. Participants were required to be enrolled in ART follow-up care at the selected referral hospitals in Northwest Ethiopia during the study period and to have documented HIV viral load results and other relevant clinical and laboratory information necessary for evaluating virological suppression. In addition, only patients with documented hepatitis B surface antigen (HBsAg)-positive status in their medical records were included in the study.

#### 2.5.2. Exclusion Criteria

Patients were excluded if their medical records lacked key information required for the analysis, including HIV viral load results, baseline CD4 cell count, WHO clinical stage, ART regimen, ART initiation date, or hepatitis B surface antigen (HBsAg) status. Patients transferred from other health facilities without complete baseline clinical and treatment history documentation were also excluded. In addition, HIV-HBV co-infected patients who were severely ill or hospitalized at the time of data collection and therefore unable to participate in the interview were excluded. Patients who had been receiving ART for less than 6 months were excluded because this duration was considered insufficient for a reliable assessment of treatment response and virological suppression.

### 2.6. Variables

#### 2.6.1. Dependent Variable

The dependent variable of this study was HIV viral suppression among adults living with HIV-HBV co-infection receiving antiretroviral therapy (ART). Viral suppression was defined as a plasma HIV viral load of <1000 copies/mL based on the most recent documented viral load result obtained after at least 6 months of ART. Participants with a viral load < 1000 copies/mL were classified as having achieved viral suppression (Yes), whereas those with a viral load ≥ 1000 copies/mL were classified as not virally suppressed (No). The outcome variable was therefore dichotomized (Yes/No) and analyzed using binary logistic regression to identify factors associated with HIV viral suppression among HIV-HBV co-infected patients.

#### 2.6.2. Independent Variables

**Socio-Demographic Variables**: These variables describe the social and demographic background of the participants and include: Age, sex, residence (urban/rural), marital status, educational level, occupation, and average family monthly income.

**Treatment-Related Variables**: These variables describe ART- and HBV-related treatment characteristics and include: Duration on ART (years), type of ART regimen, according to the Ethiopian national HIV treatment guidelines, preferred first-line ART regimens are DTG-based combinations, including TDF + 3TC + DTG and TDF + FTC + DTG. Protease inhibitor-based regimens containing lopinavir/ritonavir (LPV/r) or atazanavir/ritonavir (ATV/r) are generally used as second-line treatment following first-line treatment failure. ART treatment line was classified according to the regimen documented in the patient’s medical record and national treatment guidelines), history of ART regimen change, reason for regimen change (toxicity, failure, stock-out, etc.), treatment interruption history, use of cotrimoxazole preventive therapy (CPT), and use of isoniazid preventive therapy (IPT).

**Clinical and Laboratory-Related Variables:** These included clinical status and laboratory findings: Baseline and current CD4 cell count. WHO clinical stage (I–IV), presence of opportunistic infections, sexually transmitted infections, and other comorbidities, HIV viral load level, HBV markers (HBsAg, HBeAg status), and liver function tests (ALT, AST).

**Behavioral-Related Variables**: These variables captured patient-related behaviors that may affect treatment outcome: Alcohol consumption status, cigarette smoking status, khat chewing status, use of traditional medicine, and sexual behaviors (multiple sexual partners, condom use).

**Follow-Up and Care-Related Variables:** These variables describe patient follow-up and continuity of care: Duration of follow-up, appointment adherence, missed clinic visits, loss to follow-up, transfer out, and history of treatment interruption during follow-up.

### 2.7. Operational Definitions

**Virologic suppression (HIV):** HIV viral load result of <1000 copies/mL of plasma (or the laboratory’s detection limit) at the most recent measurement, consistent with WHO monitoring guidelines for ART response [18].

**WHO clinical stage**: Classification of HIV disease severity among adults and adolescents based on clinical signs/symptoms in resource-limited settings, according to the World Health Organization (WHO) staging system:

**Stage I**: Asymptomatic or persistent generalized lymphadenopathy [19].

**Stage II**: Mild symptoms (e.g., moderate weight loss < 10%, recurrent upper respiratory infections).

**Stage III**: Advanced symptoms (e.g., unexplained chronic diarrhea > 1-month, pulmonary TB) and/or CD4 count drop.

**Stage IV**: Severe HIV disease or AIDS-defining conditions (e.g., HIV wasting syndrome, pneumocystis pneumonia).

Duration on ART: Time in years/months since initiation of antiretroviral therapy, documented in ART records.

Treatment interruption: Any break in ART of more than 30 days without official transfer or death, as documented in patient follow-up records.

### 2.8. Data Collection Tools

Data were collected using a developed structured questionnaire and a standardized data abstraction checklist developed after reviewing national ART follow-up tools, WHO HIV/HBV management guidelines, and related scientific literature. The tools consisted of the following major components:

**Socio-Demographic Information Tool**: This section collected background information, including age, sex, residence, marital status, educational level, occupation, and monthly income. These variables were extracted from patient registration books, ART intake forms, and follow-up records.

**Treatment-Related Characteristics Tool**: This section included variables related to ART and HBV treatment such as: Type of ART regimen (Tenofovir-based or non-Tenofovir-based), Duration on ART, History of regimen change and reasons, Use of HBV-active drugs (TDF/3TC or TDF/FTC), History of treatment interruption, use of cotrimoxazole preventive therapy (CPT) and isoniazid preventive therapy (IPT) and Information was obtained from ART follow-up charts and electronic medical records.

**Clinical and Laboratory-Related Characteristics Tool**: This section documented clinical and laboratory information, including: WHO clinical stage, Baseline and most recent CD4 count, Presence of opportunistic infections, HIV viral load, HBV markers (HBsAg, HBeAg), Liver function tests (ALT, AST). These data were collected from patient cards and laboratory records.

Behavioral-Related Characteristics Tool: Behavioral factors such as alcohol consumption, cigarette smoking, khat chewing, use of traditional medicine, and sexual behaviors (e.g., multiple sexual partners, condom use) were assessed using interviewer-administered questionnaires and ART clinic records where available.

**Follow-up and Outcome Variables Tool:** This section captured follow-up information, including duration of follow-up, appointment adherence, treatment interruptions, transfer out, loss to follow-up, and mortality. Treatment outcomes such as HIV viral suppression, HBV viral suppression (if tested), and overall treatment outcome were also extracted from routine medical records.

Adherence Assessment Tool Hill-Bone Medication Adherence Scale (HBMAS): Medication adherence was assessed using a context-adapted version of the Hill-Bone Medication Adherence Scale (HBMAS). The HBMAS is a validated self-report instrument originally developed to evaluate adherence behaviors in individuals receiving long-term therapy and has been widely used in clinical and research settings. For this study, the scale was specifically adapted to assess adherence to antiretroviral therapy (ART) and HBV-active medications among adults living with HIV or HIV-HBV co-infection.

The adapted HBMAS measures three core domains of adherence: medication-taking behavior, appointment keeping, and prescription refilling. Item wording was modified to reflect ART dosing schedules, HIV clinic follow-up visits, and medication refill practices, while preserving the original structure and conceptual framework of the scale. Responses were collected using a Likert-type scale, and item scores were summed to generate a total adherence score, with lower scores indicating better adherence and higher scores indicating poorer adherence.

For analysis, medication adherence was evaluated both as a continuous outcome (total HBMAS score) and as a categorical variable. Participants were classified into good, moderate, or poor adherence based on predefined cut-off points informed by prior HBMAS applications and the score distribution within the study population. The internal consistency reliability of the adapted HBMAS was assessed using Cronbach’s alpha. Overall, the context-adapted HBMAS was considered a reliable, feasible, and appropriate tool for assessing medication adherence in the study setting.

### 2.9. Data Collection Procedures

Data were collected by trained health professionals working in ART clinics, including nurses and clinical officers who were familiar with HIV care and follow-up services. Prior to the commencement of data collection, a two-day training was provided focusing on the objectives of the study, data collection procedures, ethical considerations, confidentiality, and the proper use of data collection tools. A combination of retrospective chart review and face-to-face patient interviews was employed to obtain both clinical and socio-demographic information. Clinical and laboratory data, including ART regimen, duration on treatment, HBV status, and relevant laboratory findings, were extracted from patients’ medical records using a standardized data extraction checklist. In addition, a structured interviewer-administered questionnaire was used to collect information not available in the records, such as socio-demographic characteristics and selected behavioral factors.

A context-adapted version of the Hill-Bone Medication Adherence Scale (HBMAS) was used to measure medication adherence. The HBMAS is a widely used, simple, and reliable self-report tool for assessing adherence behaviors in chronic disease management. For this study, the scale was specifically adapted to reflect antiretroviral therapy (ART) and HBV-active medication use among adults living with HIV or HIV-HBV co-infection. The adapted HBMAS assesses key adherence domains, including medication-taking behavior, appointment keeping, and prescription refilling. Items were modified to reference ART/HBV medications and routine HIV clinic follow-up while maintaining the original structure and conceptual framework of the scale. Responses were recorded on a Likert-type scale, with higher total scores indicating poorer medication adherence. The adapted HBMAS has been previously used in clinical and research settings and was considered appropriate for identifying varying levels of medication adherence in the study population [20].

The data collection tools were developed based on a review of relevant literature. The questionnaire was initially prepared in English, translated into the local language(s), and then back-translated into English to ensure consistency of meaning. A pretest was conducted on 5–10% of the sample size in a similar health facility not included in the main study, and necessary modifications were made accordingly.

Ethical considerations were strictly observed throughout the data collection process. Confidentiality was maintained by using unique identification codes instead of personal identifiers, and all collected information was handled securely. Written informed consent was obtained from each participant prior to the interview, and interviews were conducted in a private setting to ensure comfort and privacy.

### 2.10. Quality Assurance

A range of quality assurance measures were implemented to ensure the validity, reliability, and completeness of the data collected for this study. A standardized data extraction checklist was developed based on national ART follow-up guidelines and relevant scientific literature. The tool was reviewed by experts in HIV care and public health to ensure its content validity and appropriateness for the study objectives.

Data collectors and supervisors received two days of intensive training prior to the commencement of data collection. The training covered the objectives of the study, detailed data collection procedures, ethical considerations, confidentiality, and the proper use and handling of the data collection tools.

A pretest was conducted on 5% of the total sample size using patient charts from a health facility not included in the main study. Findings from the pretest were used to refine the data extraction checklist and improve clarity, consistency, and feasibility of the data collection process.

During the data collection period, close supervision and continuous monitoring were carried out by field supervisors and the principal investigator. Completed data collection tools were reviewed daily for completeness, consistency, and accuracy. Any incomplete, inconsistent, or ambiguous entries were verified against the original source documents and corrected accordingly in consultation with the data collectors.

Data entry was performed using a double-entry system to minimize entry errors. Consistency and range checks were applied during the data cleaning process to identify and correct discrepancies. Missing values and outliers were carefully assessed and addressed appropriately during the analysis phase to ensure the integrity of the results.

To maintain uniformity across all study sites, standardized data collection procedures and tools were consistently applied in each selected health facility. This helped to ensure comparability and reduce variability in data collection practices across sites.

### 2.11. Data Processing and Analysis

Data were entered into Epi Data version 4.7.0 with programmed checks to minimize entry errors and then exported to Stata version 18 for statistical analysis. Prior to analysis, data were cleaned through systematic screening for completeness, consistency, coding errors, and missing values. Variables were labeled and coded appropriately, and data distributions were examined using graphical and statistical methods. Descriptive statistics were computed to summarize the characteristics of the study participants. Continuous variables were assessed for normality using graphical methods and statistical tests. Normally distributed variables were described using means and standard deviations, while non-normally distributed variables were summarized using medians and interquartile ranges. Categorical variables were presented using frequencies and percentages.

The magnitude of treatment outcomes, including HIV viral suppression and overall treatment success, was estimated and presented with corresponding 95% confidence intervals.

To identify factors associated with HIV viral suppression, bivariable logistic regression analysis was initially performed for each independent variable. The variables assessed included socio-demographic characteristics (age, sex, residence, marital status, and educational status), clinical and treatment-related factors (baseline CD4 count, WHO clinical stage, ART regimen, duration on ART, adherence status, opportunistic infections, and other relevant clinical and laboratory variables). Variables with a *p*-value < 0.25 in the bivariable analysis were considered candidates for inclusion in the multivariable logistic regression model. In addition, variables previously reported in the literature to be associated with HIV viral suppression, such as age and sex, were considered for inclusion irrespective of their bivariable statistical significance to control for potential confounding effects. Multivariable logistic regression analysis was then performed to identify independent predictors of viral suppression while adjusting for potential confounders. The strength of association was assessed using adjusted odds ratios (AORs) with 95% confidence intervals (CIs), and statistical significance was declared at a *p*-value < 0.05.

Model adequacy and assumptions were evaluated prior to interpretation of results. The goodness-of-fit of the final model was assessed using the Hosmer–Lemeshow test. Multicollinearity among independent variables was examined using the Variance Inflation Factor (VIF), with appropriate corrective measures taken when necessary. Additionally, potential interaction effects between key variables were explored where biologically plausible. Missing data were assessed for pattern and extent, and case-wise (complete case) analysis was applied where appropriate. Sensitivity analyses were conducted to evaluate the robustness of the findings. The final results were presented using tables, figures, and narrative summaries to facilitate clear interpretation and comparison of findings.

## 3. Result

### 3.1. Socio-Demographic Information of ART Patients

A total of 402 adult HIV-HBV co-infected patients on antiretroviral therapy were included in this study, giving a response rate of 95%. The mean age of the participants was 38.0 ± 8.9 years, with ages ranging from 18 to 68 years. The majority of participants, 140 (34.8%), were aged between 41 and 50 years. More than half of the participants were male, 235 (58.46%). With respect to educational status, 127 (31.59%) had no formal education, 111 (27.61%) had primary education, and 96 (23.88%) had attained secondary and above education (Table 1).

### 3.2. Clinical and Laboratory-Related Characteristics

Among the 402 adult HIV-HBV co-infected patients enrolled in the study. At the last follow-up, 159 (39.55%) participants had a CD4 count < 200 cells/mm^3^, 80 (19.90%) had CD4 counts between 200 and 500 cells/mm^3^, and 163 (40.55%) had CD4 counts > 500 cells/mm^3^. Regarding WHO clinical staging at ART initiation,32 (7.96%) of the study participants was classified as WHO stage I, while 169 (42.04%), 139 (34.58%), and 62 (15.42%) were in WHO stage II, III, or IV, respectively. A history of opportunistic infections was reported/documented in 111 (27.61%) of the participants, with tuberculosis being the most common comorbidity (Table 2).

### 3.3. Treatment and Follow-Up-Related Characteristics

Participants had a median ART duration of 7 years (IQR: 5–10 years). Most participants, 271 (67.41%), had been on ART for more than 5 years, whereas 131 (32.59%) had been on treatment for 5 years or less. Regarding the current ART regimen, the majority of participants, 104 (25.87%), were receiving a Dolutegravir-based regimen (ABC-3TC-DTG(5M), which has activity against both HIV and HBV. (Table 3).

### 3.4. Behavioral-Related Characteristics

Among the 402 adult HIV-HBV co-infected patients included in the study, 241 (59.95%) reported a history of alcohol consumption. Regarding cigarette smoking, 87 (21.64%) of participants had a history of smoking. With respect to khat chewing, 128 (31.84%) of participants reported current or previous khat use (Table 4).

### 3.5. Magnitude of Viral Suppression

Among the 402 HIV-HBV co-infected adults on antiretroviral therapy included in this study, 138 (34, CI, 30–39%) achieved HIV viral suppression, while 264 (66%) had an unsuppressed viral load at the time of assessment.

### 3.6. Multivariable Binary Logistic Regression Analysis

In the bivariate logistic regression analysis, medication adherence, ART regimen, duration on ART, functional status, missed appointments, and WHO clinical stage were associated with HIV viral suppression at a significance level of *p* < 0.25 and were therefore included in the multivariable logistic regression model. Good medication adherence (COR = 5.98, 95% CI: 3.41–9.77), use of the TDF-3TC-LPV/r regimen (COR = 2.51, 95% CI: 1.40–3.91), ART duration > 5 years (COR = 2.26, 95% CI: 1.33–3.38), bedridden functional status (COR = 0.31, 95% CI: 0.15–0.56), missed appointments (COR = 0.61, 95% CI: 0.35–0.92), and WHO stage IV disease (COR = 0.55, 95% CI: 0.22–1.08) showed crude associations with viral suppression. Baseline CD4 count and residence did not meet the inclusion criterion (*p* ≥ 0.25) and were therefore excluded from the multivariable analysis.

To identify factors independently associated with viral suppression, a multivariable binary logistic regression analysis was performed. After adjusting for potential confounders, medication adherence, ART regimen, duration on ART, functional status, and missed appointments remained significantly associated with viral suppression, whereas WHO clinical stage was no longer statistically significant.

Participants with good medication adherence were 5.77 times more likely to achieve viral suppression compared with those with poor adherence (AOR = 5.77; 95% CI: 3.41–9.77; *p* = 0.001). Participants receiving the TDF-3TC-LPV/r regimen had higher odds of viral suppression than those receiving the TDF-3TC-DTG regimen (AOR = 2.34; 95% CI: 1.40–3.91; *p* = 0.001). Similarly, participants who had been on ART for more than five years were 2.12 times more likely to achieve viral suppression than those on ART for less than five years (AOR = 2.12; 95% CI: 1.33–3.38; *p* = 0.002).

Regarding functional status, bedridden participants had 71% lower odds of achieving viral suppression compared with those who were working (AOR = 0.29; 95% CI: 0.15–0.56; *p* = 0.001). In addition, participants who missed clinic appointments had 43% lower odds of viral suppression than those who did not miss appointments (AOR = 0.57; 95% CI: 0.35–0.92; *p* = 0.021).

Multicollinearity was assessed using variance inflation factors (VIFs). All predictor variables had VIF values ranging from 1.03 to 1.16, indicating no evidence of multicollinearity. The Hosmer–Lemeshow goodness-of-fit test demonstrated an excellent fit of the final model (*p* = 0.94) (Table 5).

## 4. Discussion

This study found a low HIV viral suppression rate (34%) among HIV-HBV co-infected adults on ART, far below the UNAIDS 95% target [6], indicating major gaps in treatment outcomes. Globally, viral suppression rates above 80% have been reported among individuals on ART [15,21], though HIV-HBV co-infection is linked to poorer outcomes due to immune suppression, liver disease, and drug interactions [22]. In sub-Saharan Africa, suppression rates typically range from 60% to 85% [23,24] but are lower among co-infected patients [25]. Similarly, in Ethiopia, suppression rates of 70% to 88% have been reported in the general HIV population [26,27], while co-infected individuals tend to have 13.5% outcomes [28].

The high unsuppressed rate in this study may be due to poor adherence, drug resistance, suboptimal HBV-active regimens, and impaired immune recovery associated with HBV co-infection [22].

After adjustment, ART regimen, adherence, functional status, missed appointments, and ART duration were significant predictors of viral suppression. This is consistent with global evidence showing adherence and effective ART regimens as key determinants of suppression [1,15]. Poor adherence and missed visits increase virological failure due to treatment interruption [29].

Globally, optimized regimens like tenofovir-based and longer ART duration improve suppression, while better functional status supports treatment success [30]. In sub-Saharan Africa, similar factors especially adherence and retention in care are major predictors of viral suppression [23,31]. In Ethiopia, studies also confirm that good adherence, longer ART duration, and consistent follow-up improve outcomes, while poor functional status and missed visits increase failure risk [26,32,33]. These results demonstrate that in order to get the best virological outcomes, clinical and treatment-related factors predominately important for viral suppression achievement.

HIV-HBV co-infected participants with good adherence were 5.54 times more likely to achieve viral suppression than those with poor adherence, consistent with evidence across global, African, and Ethiopian settings identifying adherence as the strongest predictor of virological success. Globally, optimal ART adherence (≥95%) is associated with a 3–8-fold increase in viral suppression [6,15]. In sub-Saharan Africa, adherence improves suppression by approximately 2–6 times, despite structural and socioeconomic barriers [7], while in Ethiopia, similar magnitudes (3–6 times) have been reported [9]. This strong association is explained by the ability of good adherence to maintain adequate drug levels for continuous suppression of both HIV and HBV, prevent drug resistance, support immune recovery, and enhance treatment engagement through regular follow-up and counseling.

Another important predictor of viral suppression in this study was the ART regimen; individuals on TDF-3TC-LPV/r were 2.34 times more likely to achieve viral suppression than those on the reference regimen. This finding is consistent with global evidence, where protease inhibitor (PI)-based regimens such as lopinavir/ritonavir demonstrate strong virological efficacy due to their high genetic barrier to resistance, particularly in treatment-experienced or co-infected patients [15,34]. In sub-Saharan Africa, PI-based second-line regimens have been associated with improved viral suppression compared to failing first-line therapies, despite adherence and resource challenges [15]. Similarly, in Ethiopia, studies report better virological outcomes among patients on PI-based regimens, including LPV/r, especially after first-line failure [27]. This association is likely explained by the potency and resistance profile of LPV/r, the dual anti-HIV/HBV activity of TDF and 3TC, and enhanced clinical follow-up typically provided to patients on second-line regimens, all of which contribute to improved viral suppression outcomes.

Individuals who received antiretroviral therapy (ART) for a longer duration were 2.09 times more likely to achieve viral suppression compared to those on shorter treatment, consistent with evidence from global, African, and Ethiopian studies. Globally, prolonged ART exposure is strongly linked with sustained viral suppression as it allows adequate time for viral load reduction and immune recover [1,35]. In sub-Saharan Africa, longer duration on ART is associated with improved viral suppression, although interruptions and adherence challenges can still affect outcomes [1,15]. Similarly, in Ethiopia, evidence shows that extended time on ART improves viral suppression due to better treatment stabilization and adherence over time [9]. This relationship is explained by progressive viral load reduction, immune recovery (CD4 improvement), increased patient adherence experience, and sustained clinical monitoring, which together enhance long-term treatment success.

In this study, functional status was a significant predictor of viral suppression; individuals who were bedridden had a 71% lower likelihood of achieving viral suppression compared to those who were working. This finding is consistent with global evidence showing that poor functional or clinical status is associated with reduced treatment success due to advanced disease and limited treatment tolerance [15]. In sub-Saharan Africa, studies report that patients with impaired functional status (ambulatory or bedridden) are less likely to achieve viral suppression, often due to late presentation, comorbidities, and adherence challenges [1]. Similarly, in Ethiopia, research indicates that patients with poor functional status have lower viral suppression rates compared to working individuals [9]. This association may be explained by advanced HIV disease, opportunistic infections, reduced ability to adhere to treatment, poor nutritional status, and increased healthcare dependency, all of which negatively affect treatment outcomes.

In this study, missed clinic appointments were negatively associated with viral suppression; participants who skipped visits had a 43% lower likelihood of achieving viral suppression compared to those who attended regularly. This is consistent with global evidence showing that retention in care and regular follow-up are critical for achieving and sustaining viral suppression [1,15]. In sub-Saharan Africa, missed appointments have been strongly linked to poor adherence and virological failure due to barriers such as transportation costs, stigma, and health system constraints [7]. Similarly, in Ethiopia, studies report that patients who miss clinic visits are less likely to achieve viral suppression because of interrupted medication access and reduced clinical monitoring [9]. This association can be explained by missed opportunities for drug refills, adherence counseling, and early detection of treatment failure, all of which are essential for maintaining consistent drug levels and achieving optimal treatment outcomes.

After adjustment, factors such as sex, marital status, occupation, alcohol and drug use, stigma, social support, and HIV disclosure were not independently associated with viral suppression, suggesting that clinical and programmatic factors like adherence, regimen, and retention in care may have a stronger direct effect. This aligns with global evidence showing that once adherence and treatment-related variables are controlled for, many socio-demographic and behavioral factors lose statistical significance [1,15]. In sub-Saharan Africa, similar findings indicate that variables like stigma or social support often influence viral suppression indirectly through adherence and retention, rather than as independent predictors [7]. Likewise, in Ethiopia, studies report that although these factors may affect care engagement, they are not consistently independent predictors after multivariable adjustment [9]. This may be explained by mediation effects, measurement variability, and overlapping influences, where proximal factors such as medication adherence and consistent follow-up ultimately determine virological outcomes.

## 5. Limitations of the Study

This study has several important limitations. The cross-sectional design limits the ability to establish causal relationships between the identified factors and viral suppression. The use of secondary routine clinical data may have resulted in incomplete records and potential information bias, and some relevant variables, such as stigma, substance use, and social support, may have been incompletely documented or underreported. In addition, adherence was assessed using the HBMS tool, which relies on participants’ self-reported information and may be subject to recall and social desirability biases, potentially leading to overestimation or underestimation of adherence levels. The study was conducted in selected facilities in Northwest Ethiopia, which may limit the generalizability of the findings to other settings. Moreover, important factors such as HIV drug resistance and socioeconomic characteristics were not assessed and may have influenced the observed associations. Additionally, HBV-DNA levels and HBeAg status were not available because these tests could not be routinely performed during the study period due to reagent shortages. As a result, active HBV replication, HBV treatment response, and their relationship with HIV viral suppression could not be comprehensively evaluated. The absence of an HIV mono-infected comparison group limited our ability to assess the independent effect of HBV co-infection on HIV viral suppression.

## 6. Conclusions

This study showed that HIV viral suppression among adults with HIV-HBV co-infection in Northwest Ethiopia remains suboptimal, highlighting persistent gaps in treatment outcomes. Good adherence, longer duration on ART, use of effective ART regimens, better functional status, and regular clinic attendance were positively associated with viral suppression, whereas poor adherence, missed appointments, and advanced clinical condition reduced the likelihood of achieving HIV virological success. These findings underscore the need for policy and programmatic interventions that strengthen adherence counseling, expand differentiated service delivery models, improve patient retention and appointment-tracking systems, and ensure timely access to effective ART regimens. Prioritizing routine viral load monitoring and targeted support for clinically vulnerable patients within Ethiopia’s ART program could improve viral suppression, reduce disease burden, and accelerate progress toward national HIV and HIV-HBV co-infection treatment targets.

## 7. Clinical Implication of the Study

This study highlights several important clinical implications for managing HIV-HBV co-infected adults. First, adherence to ART should be prioritized as a core clinical intervention, with routine adherence assessment and targeted counseling integrated into every patient visit. Second, clinicians should optimize ART regimens, favoring tenofovir-based combinations that are effective against both HIV and HBV, and promptly switch regimens in cases of treatment failure. Third, early identification of patients at risk of poor outcomes such as those with poor functional status or frequent missed appointments is essential, with intensified follow-up and support provided to these groups. Additionally, retention in care strategies, including appointment tracking and patient reminders, should be strengthened to ensure continuity of treatment. Regular viral load monitoring is crucial for timely detection of non-suppression and intervention. Overall, the findings support a patient-centered, integrated care approach that combines adherence support, effective regimen management, and continuous monitoring to improve viral suppression outcomes in HIV-HBV co-infected individuals.

## Figures and Tables

**Table 1:** Socio-demographic characteristics of HIV-HBV co-infection viral suppression among adult people living with HIV-HBV co-infection in Northwest Ethiopia, 2025 (n = 402).

Variables	Category	Frequency (N)	Percentage (%)
**Sex**	Male	235	58.46
	Female	167	41.54
**Age**	18–25	38	9.45
	26–30	62	15.42
	31–40	79	19.65
	41–50	140	34.83
	51–65	83	20.65
**Marital**	Single	136	33.83
	Married	127	31.59
	Divorced	139	34.58
**Religion**	Orthodox	199	49.50
	Muslim	145	36.07
	Protestant	58	14.43
**Education**	No formal education	127	31.59
	Primary education	111	27.61
	Secondary education	96	23.88
	Higher education and above	68	16.92
**Occupation**	Government employee	29	7.21
	NGO	48	11.94
	Private organization employee	47	11.69
	Military/Police	107	26.62
	Merchant/Trader	116	28.86
	Daily labour	55	13.68
**Residence**	Urban	231	57.46
	Rural	171	42.54

**Table 2:** Clinical characteristics of HIV-HBV co-infection viral suppression among adult people living with HIV-HBV co-infection in Northwest Ethiopia, 2025 (n = 402).

Variables	Category	Frequency (N)	Percentage (%)
**Baseline CD4 count**	<200	159	39.55
	200–500	80	19.90
	>500	163	40.55
**Baseline Viral load**	<1000 copies/mm^3^	171	42.54
	≥1000 copies/mm^3^	231	57.46
**WHO clinical stage**	Stage I	32	7.96
	Stage II	169	42.04
	Stage III	139	34.58
	Stage IV	62	15.42
**Weight in kg**	≤50 kg	190	47.26
	>50 kg	212	52.74
**BMI**	<18.5 kg/m^2^	121	30.10
	18.5–24.9 kg/m^2^	131	32.59
	≥29.9 kg/m^2^	150	37.31
**Functional Status**	Working	216	53.73
	Ambulatory	138	34.33
	Bedridden	48	11.94
**TB**	Yes	111	27.61
	No	291	72.39
**Other OIs**	Yes	111	27.61
	No	291	72.39
**Admission history**	Yes	124	30.85
	No	278	69.15
**Liver function tests**	Normal (≤40 U/L)	216	53.73
	Elevated (>40 U/L)	186	46.27

**Table 3:** Treatment-related characteristics of HIV-HBV co-infection viral suppression among adult people living with HIV-HBV co-infected in Northwest Ethiopia, 2025 (n = 402).

Variables	Category	Frequency (N)	Percentage (%)
**CPT**	Yes	240	59.70
	No	162	40.30
**IPT**	Yes	231	57.46
	No	171	42.54
**Current treatment status**	First line	256	63.68
	Second line	146	36.32
**Second-line ART doses per day**	Once	151	37.56
	Twice	251	62.44
**ART Regimen**	AZT-3TC-LPV/r	62	15.42
	AZT-3TC-ATV/r	84	20.90
	TDF-3TC-LPV/r	58	14.43
	TDF-3TC-ATV/r	44	10.95
	ABC-3TC-LPV/r	50	12.44
	ABC-3TC-DTG(5M)	104	25.87
**Regimen change**	Yes	103	25.62
	No	299	74.38
**Treatment switches**	Yes	122	30.35
	No	280	69.65
**HIV Disclosure status**	Yes	175	43.53
	No	227	56.47
**Duration of ART**	<5 years	131	32.59
	>5 years	271	67.41
**Retention in care**	In care	209	51.99
	Lost follow-up	79	19.65
	Transfer out	114	28.36
**Drug adherence**	Good	233	57.96
	Poor	169	42.04
**Current Viral load result(s)**	<1000 copies/mm^3^	239	59.45
	≥1000 copies/mm^3^	163	40.55
**Current CD4 count over time**	≤200 cells/mm^3^	169	42.04
	>500 cells/mm^3^	233	57.96

**Table 4:** Behavioral-related characteristics of HIV-HBV co-infection viral suppression among adult people living with HIV-HBV co-infected in North-West Ethiopia, 2025 (n = 402).

Variables	Category	Frequency (N)	Percentage (%)
**Alcohol use**	Yes	241	59.95
	No	161	40.05
**Khat use**	Yes	128	31.84
	No	274	68.16
**Smoking**	Yes	87	21.64
	No	315	78.36
**Social support**	Yes	220	54.73
	No	182	45.27
**Stigma**	Yes	92	22.89
	No	310	77.11
**Multiple sexual partners**	Yes	89	22.14
	No	313	77.86
**Traditional medicine use**	Yes	154	38.31
	No	248	61.69

**Table 5:** Multiple regression table on magnitude and associated factors of HIV-HBV co-infection treatment outcome Among Adult people living with HIV-HBV co-infection in North-West Ethiopia, 2025 (n = 402).

Variable	Category	Suppressed		COR 95% CI	AOR 95% CI	*p*-Value
		Yes	No			
**Medication adherence**	Good	102	112	5.98 (3.41–9.77)	5.77 (3.41–9.77)	**0.001**
	Poor	36	152	1	1	
**ART regimen**	TDF-3TC-DTG	38	68	1	1	
	TDF-3TC-LPV/r	28	88	2.51 (1.40–3.91)	2.34 (1.40–3.91)	**0.001**
	AZT-3TC-LPV/r	15	46	1.21 (0.67–1.99)	1.15 (0.67–1.99)	0.606
	AZT-3TC-ATV/r	12	29	1.46 (0.58,3.67)	0.01 (0.01,1.16)	0.101
	TDF-3TC-ATV/r	20	13	0.37 (0.20,0.70)	0.28 (0.09,1.86)	0.126
	ABC-3TC-LPV/r	25	20	1.19 (0.61,2.35)	1.86 (0.17,2.43)	0.131
**Duration on ART**	<5 years	36	144	1	1	
	≥5 years	102	120	2.26 (1.33–3.38)	2.12 (1.33–3.38)	**0.002**
**WHO clinical stage**	Stage I	56	48	1	1	
	Stage II	40	54	1.35 (0.74–2.27)	1.29 (0.74–2.27)	0.368
	Stage III	29	98	1.10 (0.59–1.87)	1.06 (0.59–1.87)	0.854
	Stage IV	13	64	0.55 (0.22–1.08)	0.49 (0.22–1.08)	0.077
**Functional status**	Working	79	40	1	1	
	Ambulatory	39	101	0.91 (0.53–1.48)	0.89 (0.53–1.48)	0.640
	Bedridden	20	123	0.31 (0.15–0.56)	0.29 (0.15–0.56)	**0.001**
**Missed appointments**	Yes	32	120	0.61 (0.35–0.92)	0.57 (0.35–0.92)	**0.021**
	No	106	144	1	1	
**Baseline CD4**	Continuous	138	264	1.00 (10.99–1.00)	1.001 (0.99–1.00)	0.133
**Residence**	Urban	106	140	1.09 (0.70–1.69)	1.06 (0.68–1.66)	0.782
	Rural	32	124	1	1	

## Data Availability

The quantitative data generated and analyzed during the current study are not publicly available due to the sensitive nature of HIV-related information and the need to protect participants’ privacy and confidentiality. However, de-identified data may be made available from the corresponding author upon reasonable request and with appropriate ethical approvals from the relevant institutional review boards.

## Abbreviations

3TC, Lamivudine, ABC-3TC-DTG (5M), Abacavir + Lamivudine + Dolutegravir (5 Months Multi-month Dispensing), ABC-3TC-LPV/r, Abacavir + Lamivudine + Lopinavir/ritonavir, ALT, Alanine Aminotransferase, AOR, Adjusted Odds Ratio, ART, Antiretroviral Therapy, AST, Aspartate Aminotransferase, AZT-3TC-ATV/r, Zidovudine + Lamivudine + Atazanavir/ritonavir, AZT-3TC-LPV/r, Zidovudine + Lamivudine + Lopinavir/ritonavir, CD4, Cluster of Differentiation 4, COR, Crude Odds Ratio, CPT, Cotrimoxazole Preventive Therapy, DNA, Deoxyribonucleic Acid, FTC, Emtricitabine, HBeAg, Hepatitis B e Antigen, HBMAS, Hill-Bone Medication Adherence Scale, HBsAg, Hepatitis B Surface Antigen, HBV Hepatitis B Virus, HIV, Human Immunodeficiency Virus, IPT, Isoniazid Preventive Therapy, IQR, Interquartile Range, NGO, Non-Governmental Organization, PLHIV, People Living with Human Immunodeficiency Virus (HIV), STATA, Statistics and Data Science Software, TDF, Tenofovir Disoproxil Fumarate, TDF-3TC-LPV/r, Tenofovir Disoproxil Fumarate + Lamivudine + Lopinavir/ritonavir, TDF-3TC-LPV/r stands for Tenofovir Disoproxil Fumarate, Lamivudine, and Lopinavir/ritonavir, U/L, Units per Liter, UNAIDS, Joint United Nations Program on HIV/AIDS, VIF, Variance Inflation Factor, and WHO, World Health Organization.
